# Fusarium Graminearum Virus-1 Strain FgV1-SD4 Infection Eliminates Mycotoxin Deoxynivalenol Synthesis by *Fusarium graminearum* in FHB

**DOI:** 10.3390/microorganisms10081484

**Published:** 2022-07-23

**Authors:** Bimal Paudel, Connor Pedersen, Yang Yen, Shin-Yi Lee Marzano

**Affiliations:** 1 Department of Biology and Microbiology, South Dakota State University, Brookings, SD 57007, USA; bimal.paudel@jacks.sdstate.edu (B.P.); connor.pedersen@usda.gov (C.P.); 2 United States Department of Agriculture, Agricultural Research Service (USDA-ARS), Toledo, OH 43606, USA

**Keywords:** mycovirus, *Fusarium graminearum*, Fusarium head blight, DON, mycotoxin

## Abstract

Deoxynivalenol (DON) toxin production during the infection of *F. graminearum* in small grain crops is one of the most harmful virulence factors associated with economic losses. Metatranscriptome sequencing and RT-qPCR traced back that the only mycovirus infecting an *F. graminearum* isolate, designated as Fg-4-2, was a novel strain of Fusarium graminearum virus 1 (FgV1), designated as FgV1-SD4. The isolate Fg-4-2 showed significantly reduced virulence against wheat compared to the virus-free culture, designated as isolate Fg-4-1, which was obtained by deep freezing and single conidial germination. Notably, no DON accumulation was detected in the harvested wheat seeds infected by Fg-4-2, whereas ~18 ppm DON was detected in seeds infected by Fg-4-1. Comparison of the genome sequence of FgV1-SD4 with other identified strains of FgV1, i.e., FgV1-DK21 and FgV1-ch, indicates mutations on ORF-2 and the 3′-UTR in the genome that might be associated with hypovirulence. This mycovirus strain alone and specific genetic components of FgV1-SD4 can be further optimized to be developed as a biocontrol agent to reduce Fusarium head blight and to lower the DON accumulation levels in small grain crops due to this fungal disease.

## 1. Introduction

Mycoviruses are ubiquitous in nature, yet very few mycoviruses that exist are discovered [1,2,3] Fungal viruses are often associated with symptomless latent infections of their host; however, many mycoviruses are identified because they reduce the virulence of fungal pathogens. Cryphonectria hypovirus 1 (CHV1), which infects and reduces the virulence of *Cryphonectria parasitica*, is one of the few viruses to have been utilized as a biological control agent [4]. Differences in vegetative compatibility is a major constraint of capsidless mycoviruses such as CHV1 since they are thought to spread primarily through fungal anastomosis and within spores [5]. However, there are a few reports of the extracellular transmission of other mycoviruses, suggesting that the extracellular transmission of mycoviruses is possible [6,7]. A virus capable of extracellular transfection could potentiate the transmission of another capsidless virus by transencapsidation, as reported in *Rosellinia necatrix* [8]. Moreover, coat protein (CP)-mediated transmission of plant viruses in the families Ophioviridae and Virgaviridae by the internalization of virus particles within zoospores indicates the existence of different mechanisms of transmission of encapsidated viruses, suggesting that this phenomenon might be common in nature [9].

Fusarium graminearum virus 1 (FgV1), which infects and reduces the virulence of *Fusarium graminearum*, was isolated and characterized over a decade ago [10,11,12]. This virus under the strain name of DK21 has gained interest for its potential for being used as a biological control agent against *F. graminearum*, a notorious pathogen against small grain crops [13] causing Fusarium head blight (FHB). On the other hand, there is another reported strain of FgV1, named as FgV1-ch, that exhibits mild or no effects on fungal mycelial growth, spore production, and virulence [14], at 95.91% nucleotide identity to FgV1-DK21. FgV1 is a linear, double-stranded RNA (dsRNA) virus, which is known to be transmitted intracellularly through spores and anastomosis. The FgV1 genome contains four ORFs and is 6621 bp long without its 3′ poly (A) tail. The infection of *F. graminearum* with FgV1 has been associated with factors that relate to virulence, including reduced mycelial growth, increased pigmentation, reduced virulence on wheat plants, and reduced levels of the mycotoxin deoxynivalenol (DON) [10,11,12]. However, the underlying mechanism for how FgV1 downregulates DON biosynthesis is not clear.

Previous studies showed that dsRNA-based gene silencing machinery can be induced in response to viral infections in *Neurospora crassa* and *Cryphonectria parasitica* [15,16]. In *F. graminearum*, *Dicer-2* and *Ago-1* proteins play a critical role in small RNA-induced silencing [17]. Similarly, the role of *Dicer-like2* (*dcl2*) and *argonaute-like2* (*agl2*) as major RNAi players in this fungus is also reported [18,19,20]. Yu et al. found that FgV1 infection interferes with and downregulates *dcl2* and *ago1* to inhibit the antiviral defense mechanism of *F. graminearum* [21].

As *F. graminearum* is a major pathogen of small grain crops, viruses that induce hypovirulence and reduce the levels of the mycotoxin DON are of keen interest for their potential application as biocontrol agents. FgV1-SD4 induces hypovirulence in *F. graminearum*; however, the Chinese strain FgV1-ch does not. Narrowing down the hypovirulence determinant(s) for this virus to disarm virus-free strains has a great scope for understanding the arms race between the mycovirus and the pathogenic *F*. *graminearum*. Here, we share results of our efforts to cure this virus by single conidia germination and to explore how FgV1 infection downregulates DON biosynthesis in *F. graminearum*.

## 2. Materials and Methods

### 2.1. Plant and Fungal Materials

FHB-susceptible wheat line NIL-260-1-1-4 (NIL-S), selected from a pair of near isogenic lines of wheat carrying or not carrying *Fhb1* QTL, and FHB-susceptible Tibetan wheat landrace Y1193-6 were used in this study. The NILs were developed and kindly provided by Dr. James Anderson’s lab at University of Minnesota. Fg-SD4 isolate of *F. graminearum* was collected by Dr. Saukhat Ali’s lab at South Dakota State University from Watertown, South Dakota. *Fusarium graminearum* isolates Fg-SD-4-1 (Fg-4-1 hereafter) and Fg-SD-4-2 (Fg-4-2 hereafter) derived from the Fg-SD4 isolate were used in this study. For each experiment, 15 plants per treatment were grown in pots filled with Miracle Growth Potting Mix in a greenhouse or a growth chamber under a 16/8 h light/dark period, and 25/16 °C day/night temperature.

### 2.2. Confirmation of FgV1

After the notice of a slow growing strain of *F. graminearum*, total RNA was extracted using RNeasy mini kit (Qiagen, Valentia, CA, USA) using a bead-beating method (Mini bead beater, Biospec, Bartlesville, OK, USA). After removing the genomic DNA using TURBO-DNase (Invitrogen, Waltham, MA, USA), 1 μg of total RNA was depleted of rRNAs with the Ribo-Zero plant kit, from which a library was prepared with a ScriptSeq RNA sample preparation kit (Illumina, San Diego, CA, USA). The library was sent on ice for pair-end sequencing on an Illumina 4000 through the W. M. Keck Center of University of Illinois in Urbana-Champaign. The raw reads were deposited in the NCBI SRA database and are available under BioProject PRJNA847934. Using the same approach described earlier [22], sequence reads were assembled and blasted against customized amino acid database of viral amino acid sequences to obtain a full genome of FgV1. The FgV1 contig was traced back to strain Fg-4-2 by RT-qPCR and was absent in Fg-4-1. The sequence is deposited under GenBank accession number ON759208. The sequences were assembled to obtain the complete genome of FgV1 virus, which was named as FgV1-SD4 (FgV1 virus SD4 strain). FgV1-SD4-specific primers were designed based on the genome sequence obtained.

### 2.3. Comparison of Pathogenicity between Fg-4-1 and Fg-4-2

The two *F. graminearum* isolates derived from Fg-SD4 strain were used to compare the pathogenicity of the fungus having or not having the mycovirus FgV1, respectively, in wheat. Virus-free strain of *F. graminearum*, Fg-4-1, was obtained by single conidia germination method from the frozen stock of Fg-SD4. Briefly, a single spore was germinated in a single PDA plate from the original stock culture until a virus-free growth was obtained. For the inoculation of fungus on wheat spikes, *F. graminearum* was cultured on carboxymethyl cellulose (CMC) medium for 4 days, and then spores were collected for plant inoculation. Previously described procedures by Li and Yen were used for wheat spike inoculation [23]. Briefly, *F. graminearum* spores were filtered from CMC medium through four layers of sterile cheesecloth. The tubes with filtrate were centrifuged at 3000 rpm for 10 min to settle the conidia as pellet and were re-suspended in required volume of sterile distilled water. The concentration of conidia was counted using a hemocytometer and adjusted to ~100,000 conidia/mL. The spikelet was challenged with 10 µL of water-suspension of *F. graminearum* conidia or sterile water only (as a mock control) at the stage when intensive yellow color of anthers was observed. For each treated spike, two adjacent first-flowering spikelets were inoculated to introduce a disease pressure at the level that a control FHB-resistant genotype maximally diseased at ~28 dpi (days post inoculation). The inoculated spikes were immediately covered with plastic zip-lock bags with a wet cotton ball inside for 72 h to maintain the optimal humidity and temperature to facilitate disease establishment.

For disease evaluation, Fusarium-damaged rachides (FDR) were calculated as percentage of diseased rachides of all rachides per spike and Fusarium-damaged kernels (FDK) were calculated as percentage of diseased kernels of all harvested kernels per spike. FDR data were averaged per time-point per treatment per experiment at 7, 14, 21, and 28 dpi, respectively. Similar approach was taken to analyze FDK data per treatment. Total number of kernels per spike was also counted, averaged, and compared between three treatment groups. DON content in the harvested kernels per spike was measured by the DON testing lab at University of Minnesota and analyzed for each treatment in our lab.

### 2.4. Analysis of Viral Genomic Sequences

Amino acid sequences of ORF2 were aligned to compare the mutations between hypovirulent strains, FgV1-DK21 and FgV1-SD4, and the non-hypovirulent strain, FgV1-ch, using MUSCLE [24]. The 3′UTR of FgV1-SD4 was folded using DINAMelt [25] for quickfold and mfold programs, resulting in the same secondary structure as the output.

## 3. Results

### 3.1. The Discovery of FgV1-SD4

**Figure 1:**
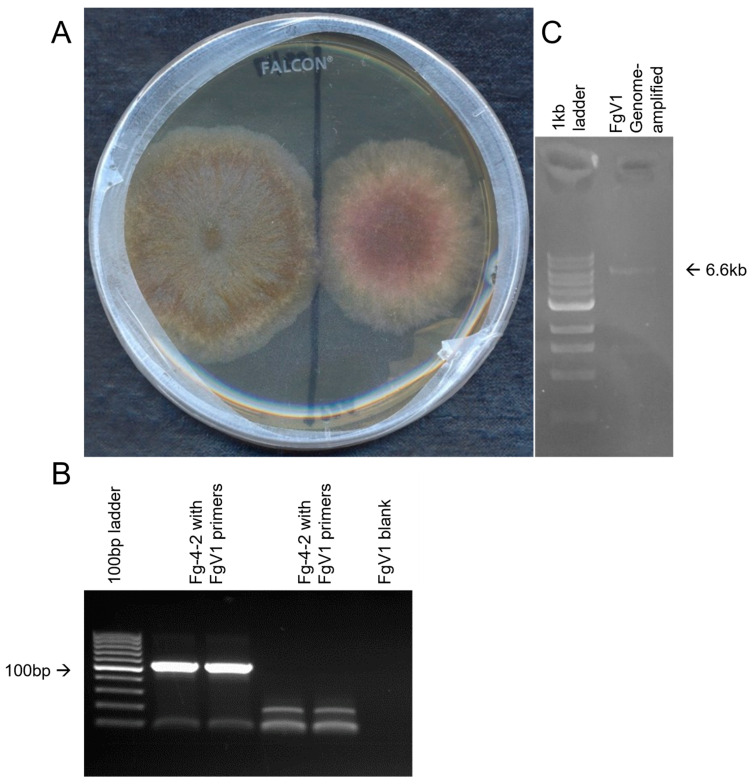
Morphological differences were observed between strains Fg-4-2 infected by FgV1-SD4 and Fg-4-1 that was cured of FgV1-SD4. FgV1-SD4 was detected by RT-PCR in strain Fg-4-2 but not in strain Fg-4-1. (**A**) Comparison of growth of Fg-4-1 (left) vs. Fg-4-2 (right) on PDA plates 5 days; (**B**) gel image confirming the detection of FgV1 viral genome in Fg-4-2 isolate of *F. graminearum*. (**C**) Amplification of the whole viral genome of FgV1 from cDNA.

First, we observed that the *F. graminearum* isolate (now named as Fg-4-2) we were using, cultured from the frozen stock of Fg-SD4 without single conidial isolation, began growing slower, showing a pinkish color, and becoming much less pathogenic than before. A comparison of this isolate with a freshly made isolate from the original frozen stock of the strain Fg-SD4, after the single conidial culture method, confirmed the differences (Figure 1A). Therefore, the virus-free strain was named Fg-4-1. Fg-4-2 was found to produce more pigments and conidia than the Fg-4-1 strain when the conidia were counted after four days of incubation. We suspected that mycovirus infection might be the cause of these difference. Fg-4-2 submitted for metatranscriptomic sequencing discovered that Fg-4-2 only harbored a novel strain of Fusarium graminearum virus 1 (FgV1-SD4), and RT-PCR showed that Fg-4-1 is free of FgV1 infection.

### 3.2. Detection and Confirmation of FgV1-SD4

RNA-seq analysis of Fg-4-2 total RNA revealed that Fg-4-2 contains a strain of mycovirus FgV1. The de novo assembled contig obtained was blasted against the viral protein database downloaded from NCBI and the FgV1 genome was the only mycovirus traced back to Fg-4-2. The FgV1 revealed in Fg-4-2 was found to be ~96% identical to FgV1-DK21 sequence-wide, and thus is named FgV1-SD4. Primers specific for the FgV1-SD4 genome were designed (Table 1) and used to detect and confirm the presence of the FgV1-SD4 genome. Using FgV1-F and FgV1-R primers on the cDNA synthesized using the FgV1-R2 reverse primer and total RNA, the presence of the FgV1-SD4 genome was confirmed in the Fg-4-2 strain of *F. graminearum* (Figure 1B), and the whole genome was amplified from cDNA using the corresponding sequences (Figure 1C). Primers designed for a novel *Fusarium* Mitovirus (FgMV-F and FgMV-R) discovered in the same metatranscriptome tested negative with RT-PCR for their presence in the isolates, supposedly belonging to other pooled *F. oxysporum* isolates. When we performed the RT-PCR on cDNA from the Fg-4-1 strain, we could not detect the FgV1-SD4 virus (Figure 1). These results confirmed that the morphological change observed in Fg-4-2 compared to Fg-4-1 was mainly due to the FgV1-SD4 infection of the latter.

**Figure 2:**
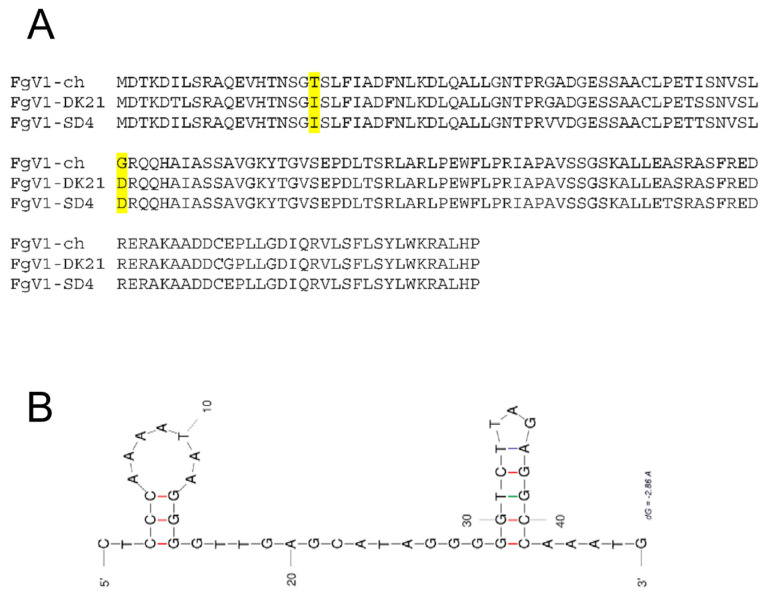
Amino acid changes in FgV1 ORF2-encoded protein (pORF2) alignment and predicted 3′-UTR secondary structure of FgV1-SD4 were speculated to explain the hypovirulence effects. (**A**) Amino acid alignment of pORF2 among FgV-1-ch, FgV1-DK21, and FgV1-SD4. (**B**) The predicted 3′-UTR (CTCCCAAAATAAGGGGTTGAGCATAGGGGGTCTTAGAGGCCAAATG) secondary structure of FgV1-SD4 with hairpin loops (UNAfold, DINAMelt quickfold, and mfold); initial ΔG = −9.00. The highlighted amino acid residues indicate the two residues different among the three reported viral variants which could be associated with different levels of hypovirulence observed.

Upon sequence analysis comparing the FgV1 ORF-2 between the hypovirulent and non-hypovirulent strains, we noticed that residues I19T and D41G were the same for the hypovirulent FgV1-DK21 and FgV1-SD4 strains but were different for the non-hypovirulent FgV1-ch strain (Figure 2A). In addition, the 3′-UTR was speculated to be the possible origin of hypovirulence [14], and the predicted RNA secondary structure shows that strain FgV1-SD4 has a different detailed structure, although still with two stem-loops (Figure 2B).

### 3.3. Pathogenicity in FHB

**Figure 3:**
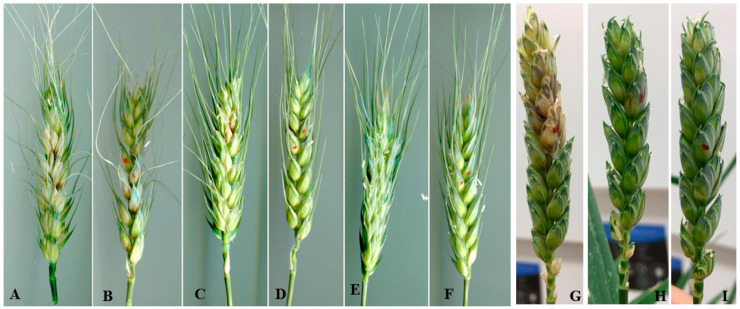
Photos showing typic symptoms of FHB on spikes of wheat genotype NIL-S (**A**–**F**) and Y1193-06 (**G**–**I**) one (in case of Y1193-06) or two weeks (in case of NIL-S) post inoculation with the *F. graminearum* isolate Fg-4-1 (**A**,**B**,**G**), Fg-4-2 (**C**,**D**,**H**), or water (**E**,**F**,**I**). Red spots indicate the inoculated spikelets.

To confirm that the reduced pathogenicity of Fg-4-2 is caused by the FgV1-SD4 infection, the two strains of *F. graminearum* were, respectively, inoculated into spikes of FHB-susceptible wheat lines NIL-S and Y1193-06 to initiate FHB. Sterile water was used in the mock control treatment. The result shows that Fg-4-2 was significantly hypovirulent compared to Fg-4-1 when the FHB severity was visually observed on the treated spikes (Figure 3).

**Figure 4:**
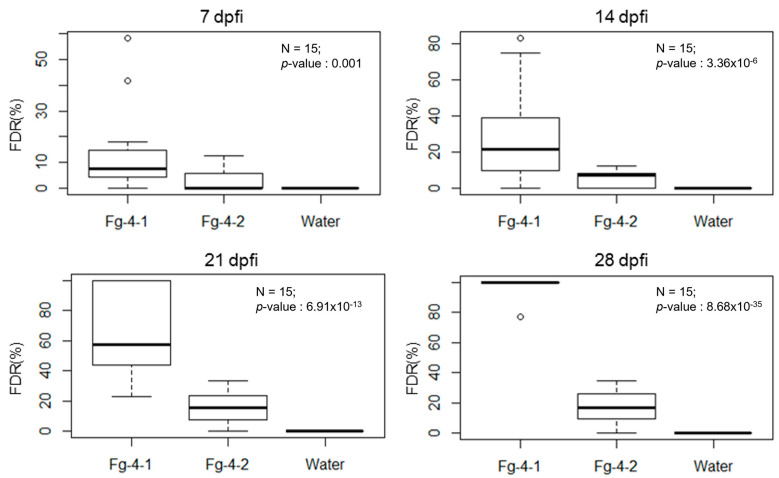
Mean FHB-damaged rachises rates (FDR) as a percentage after inoculation of wheat line NIL-S with *F. graminearum* isolates Fg-4-1, Fg-4-2, and sterile water in 7, 14, 21, and 28 dpi. Virus-free strain Fg-4-1 shows greater numbers of damaged rachises when inoculated to wheat heads at 7–28 dpi when compared to FgV1-SD4-infected strain Fg-4-2 inoculation and mock water control.

**Figure 5:**
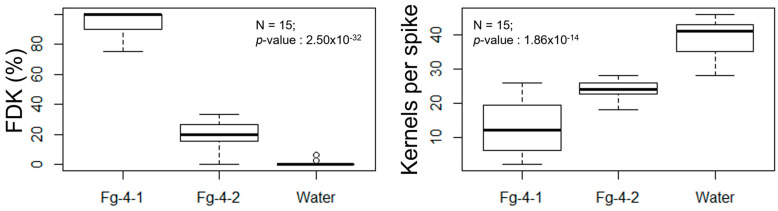
Mean FHB-damaged kernels rates (FDK) (**left**) and total kernels per spike (**right**) after inoculation of wheat NIL-S with *F. graminearum* isolates Fg-4-1 and Fg-4-2, and sterile water. Virus-free strain Fg-4-1 shows greater numbers of damaged kernels as well as decreased kernel production when inoculated to wheat heads at 7–28 dpi when compared to FgV1-SD4-infected strain Fg-4-2 inoculation and mock water control.

FDR was calculated at 7, 14, 21, and 28 days post Fusarium inoculation (dpi) with either Fg-4-1, Fg-4-2, or sterile water on the flowering spikelets (Figure 4). Our results show that FDR was significantly higher in the Fg-4-1-inoculated spikes compared to the Fg-4-2-inoculated spikes in all four time points analyzed. Similarly, FDK and the total number of kernels harvested per spike were also analyzed (Figure 5). FDK was also found to be significantly higher in the Fg-4-1-inoculated spikes compared to the Fg-4-2-inoculated ones. Additionally, the number of kernels per spike was significantly lower in the Fg-4-1-inoculated spikes compared to the Fg-4-2-inoculated spikes (Figure 5).

**Figure 6:**
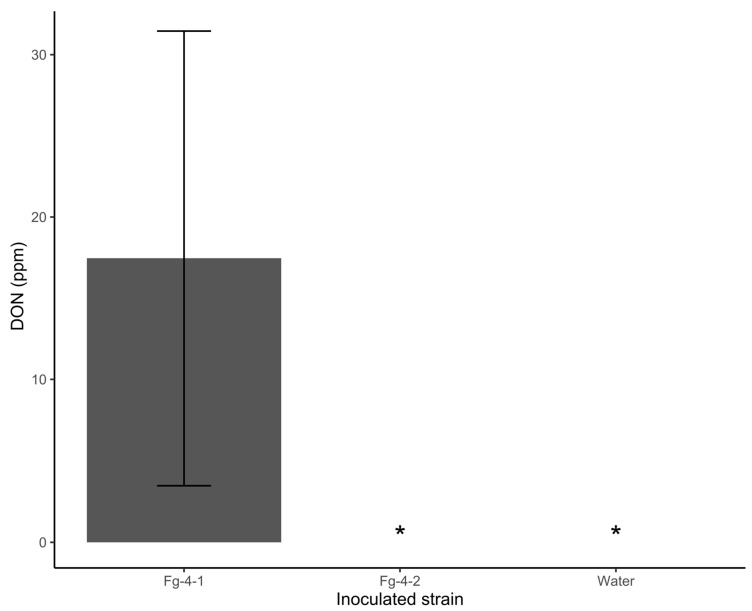
Deoxynivalenol (DON) toxin accumulation after inoculation of wheat line NIL-S with *F. graminearum* isolates Fg-4-1 and Fg-4-2, and sterile water. DON is accumulated in virus-free strain Fg-4-1; however, FgV1-SD4 infection appears to abolish DON production in strain Fg-4-2. * indicates DON undetected.

Mycotoxin DON content was also compared between kernels harvested from spikes of wheat NIL-S inoculated with Fg-4-1, Fg-4-2, and sterile water. Interestingly, no DON was detected in kernels from spikes that were inoculated with Fg-4-2, as with the water-inoculated treatment. However, there was ~18 ppm DON on seeds harvested from spikes inoculated with Fg-4-1 (Figure 6). These results demonstrate that Fg-4-2 is significantly hypovirulent compared to Fg-4-1. FgV1-SD4 reduced the pathogenicity of Fg-4-2 significantly.

## 4. Discussion

In this study, we confirmed the infection of mycovirus FgV1-SD4 in *F. graminearum* isolate-4-2 by metatranscriptomic analysis. More importantly, we validated its role in the hypovirulence of *F. graminearum* by freezing followed by single conidial culturing, allowing us to recover a virus-free culture of *F. graminearum* from Fg-4 culture. This enabled us to determine the effects of FgV1-SD4 infection on fungal morphology and pathogenicity on wheat. The observed total elimination of DON from wheat grains infected by the *F. graminearum* strain carrying FgV1-SD4 is very interesting and promising for the use of this mycovirus as a potential biocontrol agent.

RNAi-mediated defense against viral pathogens is well conserved in eukaryotic life, and it is also present in many fungi [26,27,28]. The RNA silencing response against viruses is well studied in *C. parasitica* against CHV1 and involves the induction of *dcl2* and *agl2* transcripts, and the production of hairpin RNA (hpRNA). On the other hand, CHV1 infection of *C. parasitica* suppresses the RNA silencing mechanism in the fungus through a suppressor protein known as p29, which inhibits the upregulation of *dcl2* and *agl2* in the host, to counter the host’s defense responses [16,28,29,30]. In *F. graminearum*, *Ago-1* and *Dicer-2* have critical roles in hpRNA-mediated gene silencing, and *Dicer-2* also plays a role in miRNA-like small RNA (milRNA) generation [17]. The expression of the *Dicer-1* protein was increased, but the expressions of the *Dicer-2* and *Ago-1* proteins were significantly decreased following the FgV1 infection of *F. graminearum* [19,21]. Similarly, the *Ago-1*-overexpressed *F. graminearum* mutant showed a significantly higher mycelial growth after FgV1 inoculation, compared to the wild type after FgV1 infection. The same *Ago-1* overexpression mutant also showed significantly less viral dsRNA accumulation compared to the wild type. This shows that *F. graminearum* uses its RNA silencing machinery to silence RNA viruses including FgV1. However, FgV1 overcomes this RNA silencing machinery and further suppresses the RNAi pathways of the host themselves via the suppression of *Dicer-2, Ago-1*, and *dcl2* in *F. graminearum* to establish its infection in the host by interfering with the host’s antiviral response [21].

**Figure 7:**
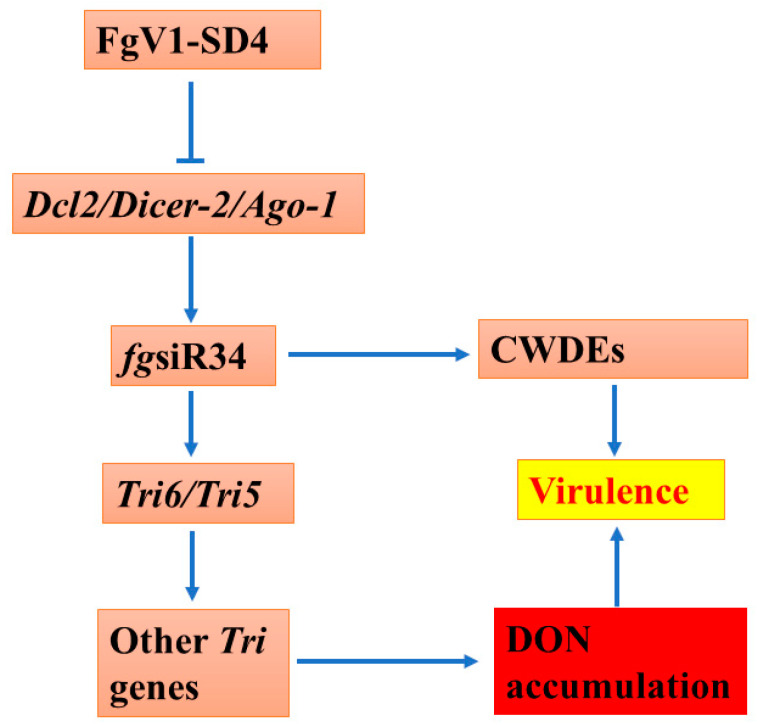
Proposed model of DON regulation in *F. graminearum* by FgV1 infection. The expression of *fg*siR34 in *F. graminearum* is indirectly suppressed by FgV1-SD4 through suppression of *Ago1/Dicer-2/dcl2* by the virus. This ultimately results in decreased biosynthesis of DON through downregulation of *Tri* genes in FHB as *fg*siR34 regulates the expression of *Tri6/Tri5* genes. *fg*siR34 also regulates expression of cell wall degrading enzymes (CWDEs) impacting the virulence of the fungus.

In the previous studies from our lab, we found that the *dcl2* knockdown mutant of *F. graminearum* showed less virulence and significantly less accumulation of DON [18]. We also found that this suppression of DON biosynthesis is probably mediated by siRNA *fg*siR34. DON biosynthesis is regulated by *Tri* genes present in three clusters in three different chromosomes of the *F. graminearum* genome. Deletion/knockdown mutant studies have shown that *Tri5*, *Tri4*, and *Tri14* are the major genes involved in DON biosynthesis, and *Tri6* and *Tri10* are the major regulatory genes of this biosynthesis pathway. Our studies show that the expression of the *Tri5* and *Tri6* genes is at least partially regulated by siRNA *fg*siR34 [18,31]. *Tri5* gene encodes an enzyme that catalyzes the first step of DON biosynthesis, and *Tri5* is controlled by *Tri6*. A potential target site that matches with the partial sequence of *fg*siR34 between the *Tri6* and *Tri5* genes in the cassette of *Tri* genes in *F. graminearum* also suggests a regulatory role of this small RNA in *Tri* genes. Here, we propose a model of DON biosynthesis reduction due to FgV1-SD4 infection in *F. graminearum* (Figure 7). We propose that the expression of *fg*siR34 in *F. graminearum* is indirectly suppressed by FgV1-SD4 through the suppression of *Ago1/Dicer-2/dcl2* by the virus. This ultimately results in the decreased biosynthesis of DON through the downregulation of *Tri* genes in FHB as *fg*siR34 regulates the expression of the *Tri6/Tri5* genes.

The comparative approach based on the ORF2 sequence alignments to the other known strains helped to narrow down the determinant of hypovirulence to two amino acid residues and/or 3′UTR folding. It will be insightful to make reverse genetics systems available to mutate these residues or swap out the 3′UTR regions to pinpoint the exact changes that explain for the very different outcomes in pathogenicity as well as DON production.

## Figures and Tables

**Table 1:** List of primers used in this chapter.

Primer Name	Sequence	Purpose
FgV1-F	GTTGCGTTGGAGGTTGACAC	RT-PCR
FgV1-R	CCAAAAACCACACGTCGTCC	RT-PCR, cDNA synthesis
FgV1-F2	GGGGTATACTCTGATTATTTGAATTT	Whole viral genome RT-PCR amplification, Sanger sequencing
FgV1-R2	CATTTGGCCTCTAGACCCCCTATGCT	Whole viral genome RT-PCR amplification, Reverse transcription, Sequencing
FgMV-F	ACCATATCCCTTTTGGGGCTG	RT-PCR
FgMV-R	GTGCTCTTCCGATCTCCGTG	RT-PCR

## Data Availability

Metagenomic raw data is available at the NCBI SRA at PRJNA847934.
